# Balancing Safety and Efficacy: Factor XIa INHIBITORS vs. DOACs in Patients with Atrial Fibrillation: A Systematic Review and Meta-Analysis of Randomized Controlled Trials †

**DOI:** 10.3390/jcm14228234

**Published:** 2025-11-20

**Authors:** Jacinthe Khater, Marco Frazzetto, Shamin Hayat Mahmud, Ashkan Yahyavi, Sara Malakouti, Bharat Khialani, Bernardo Cortese

**Affiliations:** 1 DCB Academy, 20143 Milan, Italy; jacinthekhater1234@hotmail.com (J.K.); marcofrazzetto7@gmail.com (M.F.); dr.saramalakouti@gmail.com (S.M.); bharat.v.khialani@nhghealth.com.sg (B.K.); 2 Faculty of Medical Sciences, Rafic Hariri University Campus, Lebanese University, Hadath 6573, Lebanon; 3 Harrington Heart and Vascular Institute, University Hospitals, Cleveland, OH 44106, USA; shamin.mahmud@uhhospitals.org (S.H.M.); seyedashkan.yahyavi@uhhospitals.org (A.Y.); 4 Fondazione Ricerca e Innovazione Cardiovascolare, 20143 Milan, Italy; 5 Tan Tock Seng Hospital, National Healthcare Group, Singapore 308433, Singapore

**Keywords:** atrial fibrillation, factor XIa inhibitors, direct oral anticoagulants, bleeding risk, stroke prevention

## Abstract

**Background**: Current ESC guidelines recommend the use of oral anticoagulant therapy in patients with atrial fibrillation to reduce the risk of arterial embolization. Recently, Factor XIa inhibitors were investigated as an alternative to the commonly used DOACs. **Aims:** This systematic review and meta-analysis aim to assess whether Factor XIa inhibitors lead to lower risks of bleeding compared to DOACs in patients with atrial fibrillation. **Methods:** PubMed, Cochrane, and EMBASE were searched. The primary endpoint was the occurrence of a composite of Major or clinically relevant non-major bleeding. Secondary endpoints included death from any cause, cardiovascular death, any adverse effects, any serious adverse effects, minor bleeding, stroke, or systemic embolism. **Results:** A total of 3 studies met the inclusion criteria and were included in the qualitative and quantitative analysis, comprising 16,772 patients. Factor XIa inhibitors were associated with significantly fewer major or clinically relevant non-major bleedings than DOACs (RR = 0.40 [95% CI [0.32, 0.51]), I^2^ = 3%, *p* < 0.00001). There were no differences between Factor XIa inhibitors and DOACs regarding the occurrence of death (RR = 0.83 [95% CI 0.63, 1.08]), I^2^ = 0%, (*p* = 0.15) and cardiovascular death (RR = 1.09 [95% CI 0.74, 1.63]), I^2^ = 0%, (*p* = 0.65). Interestingly, the risk of stroke and systemic embolism was higher among the patients exposed to factor XIa inhibitors compared to the DOACs group RR = 6.05 [95% CI [1.02, 35.94]), I^2^ = 5%, (*p* = 0.05). **Conclusions:** Factor XIa inhibitors are superior to DOACs in terms of bleeding complications. However, this meta-analysis shows a significantly higher risk of stroke or systemic embolism as compared to commonly used DOACs.

## 1. Introduction

Atrial fibrillation (AF) is the most common clinically significant, sustained cardiac arrhythmia. The worldwide prevalence of AF has significantly increased over the past decade, reportedly rising from 33.5 million in 2010 to over 59 million in 2019. AF is associated with increased morbidity and mortality, leading to an elevated risk of stroke, myocardial infarction, and heart failure. Systemic thromboembolic events, including stroke, occur in AF as the disorganized rhythm predisposes the formation of intracardiac thrombi due to blood stasis, which can then travel in the circulation and embolize at distant sites. Hence, anticoagulation remains fundamental in the management of AF to prevent stroke [1].

Historically, vitamin K antagonists (VKAs) such as warfarin were utilized as the primary anticoagulation in AF, as they conferred protection from thromboembolism. However, VKAs posed significant challenges due to their narrow therapeutic range, numerous pharmacologic and dietary interactions, and adverse effect profile. To overcome such pitfalls, direct oral anticoagulants (DOACs) were introduced and transformed AF management, with data showing DOACs to have similar or better efficacy for stroke prevention in AF while having fewer rates of intracranial bleeding and a favorable adverse effect profile compared to warfarin. DOACs, compared to VKAs, have a faster onset of action, fewer interactions, and require less monitoring, thus becoming the anticoagulation of choice in patients with AF and an elevated stroke risk according to the latest guidelines [2,3]. Nevertheless, limitations of DOACs include an elevated risk of significant bleeding, especially gastrointestinal bleeding, and thus create opportunities for novel anticoagulation strategies to minimize bleeding risks.

In this context, the development of Factor XI (FXI) inhibitors has emerged as a promising alternative to DOACs in finding the right balance between adequate thromboembolic protection and mitigating bleeding risks in patients with AF. FXI inhibitors reduce thrombosis without significantly impacting physiologic hemostasis, theoretically preventing pathologic thrombi formation without increasing bleeding risk [4,5].

Several randomized controlled trials (RCTs) have been performed and are currently underway to compare FXI inhibitors with DOACs in patients with AF [6,7,8]. Most of the data are from smaller Phase II, and one Phase III, RCT to establish the safety and efficacy of FXI inhibitors compared to the current standard of DOAC therapy in AF. However, there are currently no large-scale meta-analyses to evaluate FXI inhibitors in this patient population comprehensively. As the Phase II RCTs were designed to compare the safety profiles of FXI inhibitors at different doses with DOACs, these studies did not have sufficient power to evaluate clinical efficacy and outcomes. In this regard, a meta-analysis would be beneficial in synthesizing evidence and increasing the power of existing studies to determine clinical outcomes and provide a more comprehensive understanding of the utility of FXI inhibitors. Thus, this meta-analysis aims to compare the efficacy and safety of FXI inhibitors versus DOACs in patients with AF.

## 2. Materials and Methods

### 2.1. Search Strategy

This systematic review and meta-analysis was conducted in accordance with the Cochrane Handbook for Systematic Reviews of Interventions and was reported according to the Preferred Reporting Items for Systematic Reviews and meta-analyses (PRISMA) guidelines, the PRISMA checklist is provided in the Supplementary Materials. The initial search included the Cochrane Library, Embase, PubMed using the keywords: “Factor XIa inhibitors, FXIa inhibitors, Milvexian, Asundexian, Abelacimab, Direct oral anticoagulants, DOACs, Apixaban, Rivaroxaban, Edoxaban, Dabigatran, Atrial fibrillation, AF, A Fib, atrial arrhythmia, cardiac arrhythmia”. The retrieval strategy was applied with customization of search strings to accommodate the recommendations of each database. No document type or other relevant restrictions will be used in the retrieval process, and unpublished articles will be excluded. In addition, we will explore the references of the included studies to identify additional potential articles. No language restrictions were applied. The references from all included studies and previous reviews were also manually searched. Two authors independently extracted the data after predefined search criteria and quality assessment. The prospective meta-analysis protocol was registered on PROSPERO version 2.0.25 under the ID: CRD42025645812.

### 2.2. Eligibility Criteria

We only included (1) RCTs comparing Factor XIa inhibitors to DOAC therapy in adult patients of any age diagnosed with atrial fibrillation, (2) Anticoagulants prescribed through the systemic FXI targeted agents, oral or parenteral. (3) Adults ≥ 18 years, (4) Known to have A fib, (5) Indication of anticoagulation for long-term treatment of AF, (6) studies with 2 arms showing comparison of Factor XIa Inhibitors to DOACs, (7) Reported outcomes: clinically relevant non-major bleeding, death from any cause, cardiovascular death, any adverse effects, any serious adverse effects, minor bleeding, stroke, or systemic embolism, (8) English language, (9) Full-text available and available results. Furthermore, studies meeting any of the following exclusion criteria were excluded: (1) No direct comparison of FactorXIa inhibitors to DOACs, (2) An indication other than Atrial fibrillation for OACs (3) Case re-ports, reviews, editorials, abstracts (4) Animal or in vitro studies, (5) Duplicate or overlapping data.

### 2.3. Screening and Data Extraction

All the search results were imported into the Rayyan.ai software (https://www.rayyan.ai/) for the management of the review process. The screening process was carried out by two independent reviewers (JK and MF) who assessed titles and abstracts for relevance, followed by a full-text review to determine eligibility. A double-blind approach was used to minimize potential selection bias. Reviewer discrepancies were resolved through discussion or consultation with a third reviewer (BC).

### 2.4. Quality Assessment

We evaluated the risk of bias in randomized studies using version 2 of the Cochrane Risk of Bias assessment tool. Disagreements were resolved through a consensus after discussing the reasons for the discrepancy.

### 2.5. Statistical Analysis

The Data was analyzed using Review Manager (RevMan) software version 5.4. Pooled treatment effects for binary endpoints were compared using a risk ratio (RR) with a 95% confidence interval (CI). Heterogeneity was examined with the Cochran Q test and I^2^ statistics. A random-effects model was employed, and the DerSimonian and Laird method was applied to estimate the between-study variance (τ^2^). I^2^ values of 25%, 50%, and 75% were considered indicative of small, moderate, and high levels of heterogeneity, respectively.

### 2.6. Study Endpoints

The primary endpoint was the occurrence of a composite of Major or clinically relevant non-major bleeding.

Major bleeding was defined according to the trial-specific definitions, predominantly following the International Society on Thrombosis and Haemostasis (ISTH). Clinically relevant non-major bleeding (CRNMB) was defined as any overt bleeding requiring medical attention but not meeting criteria for major bleeding.

Secondary endpoints included death from any cause, cardiovascular death, any adverse effects, any serious adverse effects, minor bleeding, stroke, or systemic embolism. A qualitative synthesis of the results is provided in Table 1 and Table 2.

## 3. Results

### 3.1. Qualitative Analysis

**Figure 1:**
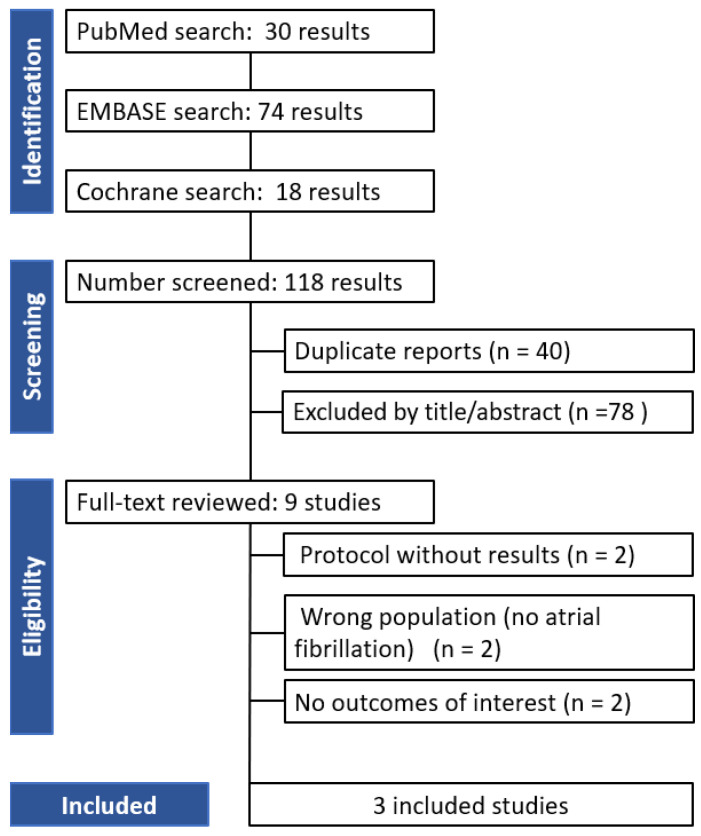
PRISMA flow diagram of study screening and selection.

A total of 9 full-text articles were assessed for eligibility. Of these, six studies were excluded for the following reasons: two were protocols with no results, two did not include the population of interest (patients with atrial fibrillation), and two did not report relevant outcomes of interest. Ultimately, 3 studies met the inclusion criteria and were included in the final analysis. The flowchart of the systematic search is illustrated in Figure 1. The most relevant clinical and procedural findings from the included articles are summarized in Table 1 and Table 2. All the studies were randomized trials. The present systematic review embraced 16,772 patients who received clinically indicated OAC therapy for atrial fibrillation: 8728 patients were allocated to continued Factor XIa inhibitor therapy and 8044 (48%) patients to DOACs. Atrial fibrillation was the indication for OAC therapy in all the included studies. Asunedexian (Factor XIa inhibitors) and Apixaban (DOACs) were used in 2 RCTs (PACIFIC-AF and OCEANIC-AF), for AZALEA TIMI 71, Abelacimab (Factor XIa inhibitors) and Rivaroxaban (DOACs) were used. The prevalence of hypertension and diabetes was approximately 85% and 36%, respectively, while a history of stroke was observed in 17.33% of the included populations.

### 3.2. Risk of Bias Assessment

The major findings of the risk-of-bias assessment are reported in Supplementary Table S1. One open-label study was considered at low risk of bias, and 2 with some concerns. One trial, the OCEANIC-AF, had some concerns about the missing outcome data and bias in the selection of reported results.

### 3.3. Quantitative Analysis

3 meta-analyses were performed to compare the safety and efficacy of Factor XIa inhibitors versus DOACs in atrial fibrillation patients. Three studies reported data on Major or clinically relevant non-major bleeding, all-cause mortality, cardiovascular mortality, any adverse or serious adverse effects, minor bleeding, and incidence of strokes. Factor XIa inhibitors were associated with significantly fewer events of major or clinically relevant nonmajor bleeding than DOACs (RR = 0.40 [95% CI 0.32, 0.51]), I^2^ = 3%, (*p* < 0.00001). There were no differences between Factor XIa inhibitors group and the DOACs group regarding the occurrence of all-cause mortality (RR = 0.83 [95% CI 0.63, 1.08]), I^2^ = 0%, (*p* = 0.15), rates of cardiovascular death (RR = 1.09 [95% CI 0.74, 1.63]), I^2^ = 0%, (*p* = 0.65), all adverse events (RR = 1.01 [95% CI 0.97, 1.04]) I^2^ = 0%, (*p* = 0.63), minor bleeding (RR = 1.01 [95% CI 0.00, 8797.00]) I^2^ = 0%, (*p* = 0.58), and risk of stroke and systemic embolism was higher among the patients exposed to factor XIa inhibitors compared to the DOACs group (RR = 6.05 [95% CI 1.02, 35.94]), I^2^ = 5%, (*p* = 0.05). The pooled relative effect for major bleeding with FXIa inhibitors versus DOACs was RR 0.40 (95% CI 0.32–0.51). With a pooled control event rate of 28.5 major bleeds per 1000 patients, this corresponds to an absolute reduction of 17.1 fewer major bleeds per 1000 patients (95% CI 14.0 to 19.4 fewer per 1000). The NNT to prevent one major bleed is 59 (95% CI 52 to 72).

**Figure 2:**
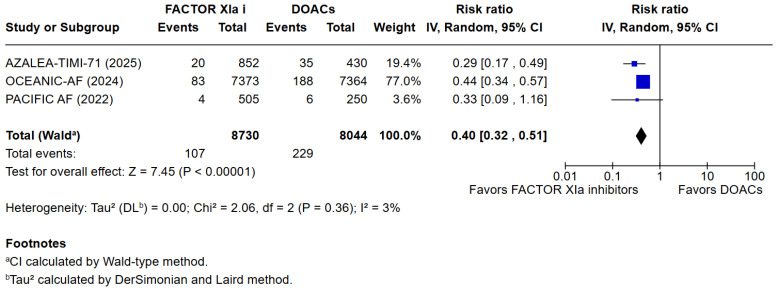
Risk ratio of major or clinically relevant non-major bleeding between the Factor XIa inhibitor group and the DOAC group.

**Figure 3:**
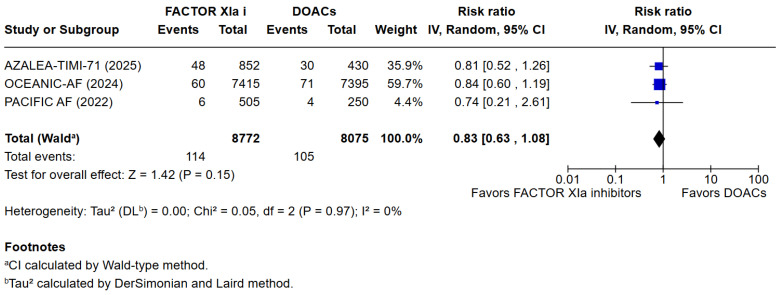
Risk ratio of all-cause death between the factor XIa inhibitor group and the DOAC group.

**Figure 4:**
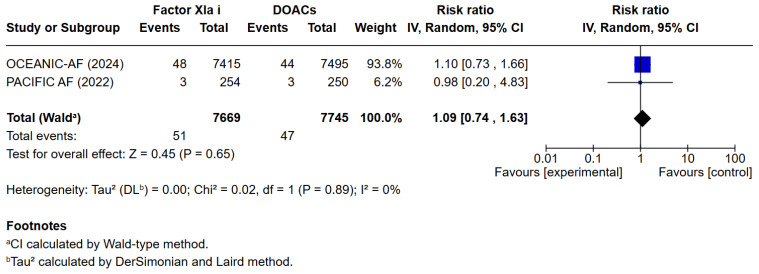
Risk ratio of cardiac death between the factor XIa inhibitor group and the DOAC group.

**Figure 5:**
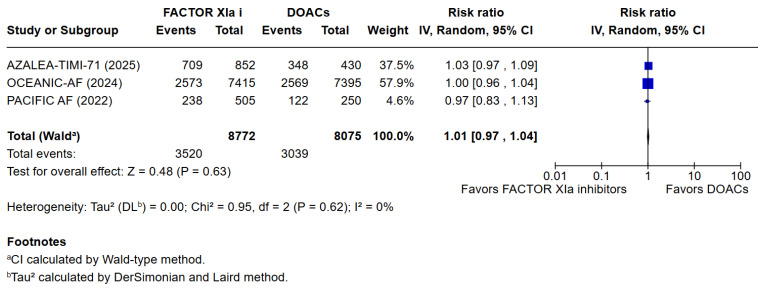
Risk ratio of any adverse effect between factor XIa inhibitors group and the DOAC group.

**Figure 6:**
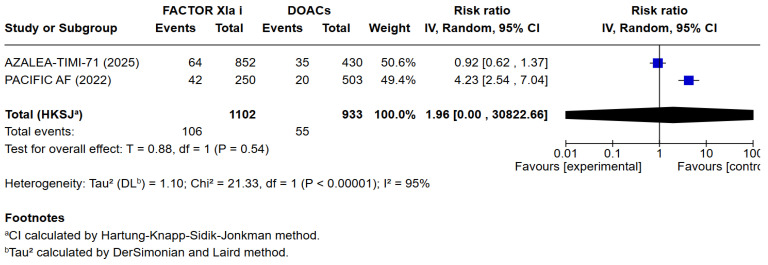
Risk ratio of any serious adverse effects between factor XIa inhibitors group and the DOAC group.

**Figure 7:**
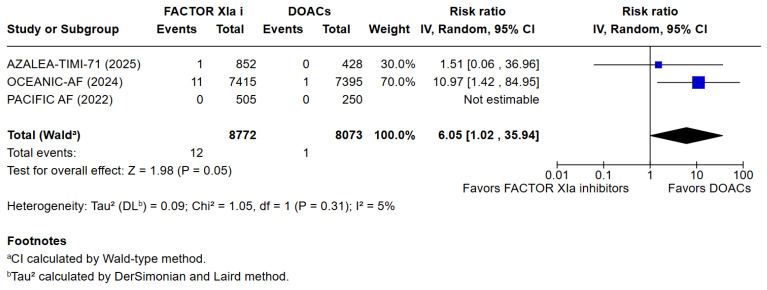
Risk ratio of stroke or systemic embolism between factor XIa inhibitors group and the DOAC group.

**Figure 8:**
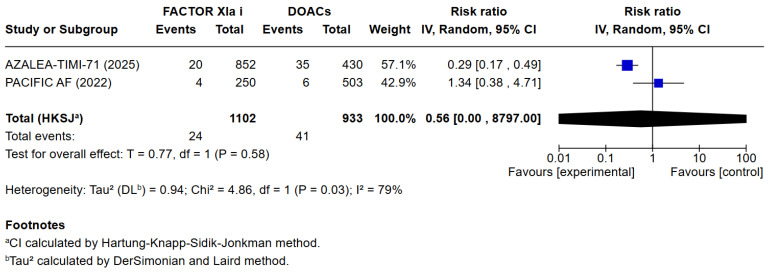
Risk ratio of minor bleeding between factor XIa inhibitors group and the DOAC group.

The results of the quantitative analysis were graphically displayed using Forest plots (Figure 2, Figure 3, Figure 4, Figure 5, Figure 6, Figure 7 and Figure 8).

## 4. Discussion

Our meta-analysis of three randomized trials (AZALEA–TIMI 71, OCEANIC-AF, PACIFIC-AF; n ≈ 16,772) reveals that targeting factor XIa (FXIa) dramatically reduces bleeding but may compromise stroke prevention compared with standard DOAC therapy [1]. Consistent with prior analyses, FXIa inhibitors (abelacimab or asundexian) significantly lowered major and clinically relevant nonmajor bleeding versus apixaban/rivaroxaban [4]. For example, AZALEA–TIMI 71 reported major/CRNM bleeding in only ~5–6% on abelacimab (both 90 mg and 150 mg) versus 15.4% on rivaroxaban [6]. PACIFIC-AF also saw fewer bleeding events with asundexian (pooled 4 vs. 6 events) despite near-complete FXIa blockade [8]. In contrast, efficacy endpoints (stroke, systemic embolism) trended worse with FXIa agents. In the large OCEANIC-AF trial, stroke or systemic embolism occurred in 1.3% of asundexian-treated patients versus 0.4% on apixaban (HR ≈ 3.8, 95% CI 2.46–5.83) [7], prompting early termination for inferiority. AZALEA noted slightly higher stroke rates on abelacimab (2.3–2.6%) than on rivaroxaban (1.6%), though event counts were very small. Thus, FXIa inhibition appears to trade some antithrombotic efficacy for markedly improved safety [6,9]. Clinicians must balance these trade-offs: DOACs exert potent stroke protection at the cost of more bleeding, whereas FXIa inhibitors may “uncouple” hemostasis from thrombosis, yielding fewer bleeds but potentially less robust stroke prevention [5]. Mechanistic insights. These results reflect the distinct biology of factor XI. FXI lies in the intrinsic (contact) pathway, amplifying thrombin generation via feedback loops, whereas DOACs (e.g., apixaban, rivaroxaban) directly inhibit factor Xa in the common pathway [10]. In normal hemostasis, tissue factor (TF)-mediated extrinsic coagulation predominates, and FXI’s role is modest [11]. By contrast, pathological thrombosis—especially under low-flow (stasis) or on artificial surfaces—relies more on FXI-driven amplification to sustain clot growth [10,11]. This difference underpins the promise of FXI inhibition: inherited FXI deficiency protects against venous thromboembolism and ischemic stroke while causing only mild bleeding tendencies [12]. Indeed, epidemiologic data show that patients with partial FXI deficiency (FXI activity ≤50%) have ~50% lower risk of cardiovascular events (adjusted HR ≈ 0.52–0.57) and ~75% lower VTE risk (HR ≈ 0.26) than those with normal FXI [12]. Animal models similarly demonstrate that blocking FXI reduces thrombosis without provoking bleeding [10]. Conceptually, FXI inhibition decouples thrombosis from hemostasis: it attenuates pathologic clotting cascades while sparing the initial platelet plug and TF-dependent thrombin burst required for normal bleeding control [5,10]. However, our findings confirm that this “uncoupling” is not absolute. The larger OCEANIC-AF trial showed that nearly complete pharmacologic FXIa inhibition (Asundexian 50 mg once daily) failed to prevent strokes as effectively as apixaban [7]. Several explanations are possible. First, even near-total FXI suppression may leave enough residual thrombin generation (via TF/FVIIa pathways or FXIIa) to allow clotting under high-risk conditions like left atrial appendage stasis. Second, some embolic events in OCEANIC-AF were non-cardioembolic, where systemic anticoagulation offers limited benefit [7]. Third, species differences and the complexity of human coagulation may mean FXI is less dominant than hoped [10]. Importantly, none of the trials showed a significant increase in hemorrhagic stroke with FXIa inhibitors [7,8,9]. Thus, the net effect is a safety-oriented strategy: FXI blockade offers a strong bleeding advantage, at the expense of a signal (yet to be fully quantified) toward higher ischemic events [9]. FXIa inhibitors differ markedly from DOACs in molecular structure, pharmacokinetics (PK), and administration. Abelacimab is a human monoclonal antibody that binds the catalytic domain of factor XI, preventing its activation [13]. It is given by subcutaneous injection once monthly, leading to sustained FXI suppression, and is eliminated via proteolytic degradation largely independent of renal clearance [14]. By contrast, rivaroxaban is orally given once daily, with ~66% renal elimination, and apixaban is given twice daily with ~27% renal elimination [14]. Asundexian and milvexian are orally active small molecules, heavily excreted via the fecal/biliary route (~80%) with minimal renal clearance, but are CYP3A4 substrates [14]. These pharmacologic properties influence clinical decision-making. For example, abelacimab’s long half-life may improve adherence but complicates reversal in the event of bleeding, while DOACs have established antidotes [14]. FXI inhibitors may also be preferable in patients with renal impairment, since most are not renally cleared [14]. Indeed, several ongoing trials are exploring FXI inhibitors in dialysis-dependent patients [13]. Currently, all major atrial fibrillation guidelines (ESC, AHA/ACC/HRS) recommend DOACs as first-line anticoagulants, with no approved FXa inhibitors 3. Our findings suggest that, if proven effective in Phase III trials, FXIa inhibitors could benefit select subgroups. Patients with high bleeding risk, prior intracranial hemorrhage, or concomitant antiplatelet therapy may particularly benefit from the improved safety profile [6,9]. Renal impairment represents another subgroup where FXIa inhibitors may be advantageous due to reduced renal clearance [14]. However, until efficacy is confirmed, guidelines will remain DOAC-centric [3]. Several caveats must be acknowledged. Only three RCTs have directly compared FXIa inhibitors and DOACs, and only one was a large-scale Phase III trial (OCEANIC-AF) [7]. AZALEA and PACIFIC were Phase II studies, underpowered for efficacy [8,9]. Follow-up was relatively short (<6 months in OCEANIC), and heterogeneity existed in study design, patient selection, and endpoint definitions [9]. Event rates for stroke were very low in PACIFIC and AZALEA, limiting precision [8,9]. Furthermore, not all FXIa agents (e.g., milvexian, osocimab) were represented, leaving important gaps. Multiple Phase III trials are ongoing. LIBREXIA-AF (milvexian vs. apixaban) is enrolling ~15,500 patients globally to test noninferiority for stroke prevention with bleeding reduction as a co-primary endpoint [15,16]. LILAC–TIMI 76 is investigating abelacimab in AF patients deemed unsuitable for standard anticoagulation [17]. Additional studies are testing FXI inhibition in venous thromboembolism, TAVR, and dialysis [16]. If these confirm favorable safety without unacceptable efficacy trade-offs, guidelines may incorporate FXI inhibitors into routine AF management, particularly for high-bleeding-risk populations.

## 5. Conclusions

This systematic review and meta-analysis suggest that Factor XIa inhibitors may offer a favorable bleeding profile compared with DOACs, with a significant reduction in major and clinically relevant non-major bleeding. However, this apparent safety advantage must be interpreted with caution, as the evidence base is still limited—comprising one phase 3 randomized trial and two smaller phase 2 studies that used different agents and comparator DOACs. Importantly, the increased incidence of stroke and systemic embolism observed with Factor XIa inhibitors, driven largely by the largest and most statistically weighted trial (OCEANIC-AF), underscores the need for prudence. The early termination of that trial due to excess ischemic events highlights that the anticoagulant efficacy of these agents remains uncertain.

Overall, while the concept of “uncoupling” thrombosis from hemostasis remains attractive, current data are insufficient to support replacing DOACs in routine atrial fibrillation management. Larger ongoing phase 3 trials such as LIBREXIA–AF and LILAC–TIMI 76 will be essential to determine whether Factor XIa inhibition can achieve comparable stroke protection with improved safety, potentially redefining future anticoagulation strategies.

## Figures and Tables

**Table 1:** Summary of the included studies.

	Author, Year	Design of the Study and Follow-Up Period	Route of Administration of the Drug	Type of OACs		Sample Size (n)		Age (years old)		Female n (%)		CHA_2_DS_2_-VASc ^a^ Score		Paroxysmal AF ^b^ n (%)		Persistent and Longstanding Persistent AF n (%)		Permanent AF n (%)	
				Factor XIa Inhibitor	DOAC ^c^	Factor XIa Inhibitor	DOAC ^c^	Factor XIa Inhibitor	DOAC ^c^	Factor XIa Inhibitor	DOAC	Factor XIa Inhibitor	DOAC	Factor XIa Inhibitor	DOAC	Factor XIa Inhibitor	DOAC	Factor XIa Inhibitor	DOAC
AZALEA TIMI 71 *	Ruff et al., 2025 [6]	RCT	Abelacimab 150 mg SC monthly vs. Abelacimab 90 mg SC monthly vs. Apixaban 20 mg OD	Abelacimab	Rivaroxaban	427	430	74 (69–78) Median (IQR ^d^)	74 (69–79) Median (IQR)	388 (45.3)	184 (42.8)	5 (4–5) Median (IQR)	5 (4–6) Median (IQR)	444 (52.2)	225 (52.6)	171 (20.1)	97 (22.7)	235 (27.6)	106 (24.8)
OCEANIC AF	Piccini et al., 2024 [7]	RCT	Asundexian 50 mg BD vs. Apixaban 5 mg BD oral	Asundexian	Apixaban	7415	7395	73.9 ± 7.7 Mean ± SD ^e^	73.9 ± 7.7 Mean ± SD	2656 (35.8)	2558 (34.6)	4.3 ± 1.3 Mean ± SD	4.3 ± 1.3 Mean ± SD	2760 (37.2)	2641 (35.7)	2209 (29.8)	2233 (30.2)	2327 (31.4)	2384 (32.2)
PACIFIC AF	Piccini et al., 2022 [8]	RCT	Asundexian 50 mg BD vs. Apixaban 5 mg BD oral	Asundexian	Apixaban	254	250	73.4 ± 8.2 Mean ± SD	74.3 ± 8.3 Mean ± SD	200 (40)	109 (44)	3.9 ± 1.3 Mean ± SD	4.1 ± 1.4 Mean ± SD	237 (47)	117 (47)	147 (29.1)	65 (26)	-	-

^a^ CHA_2_DS_2_-VASc scores range from 0 to 9, with higher scores indicating a greater risk of stroke. Congestive heart failure, hypertension, age of 65 to 74 years, diabetes, vascular disease, and female sex are each assigned 1 point; age of 75 years or older, previous stroke, transient ischemic attack, and previous thromboembolism are each assigned 2 points, ^b^ Atrial Fibrillation, ^c^ Direct-acting Oral Anticoagulant, ^d^ Interquartile Range, ^e^ Standard Deviation, * The numbers for age and CHA_2_DS_2_-VASc score are reported for the study group receiving Abelacimab 150 mg.

**Table 2:** Summary of the characteristics of the included studies.

	Trial Phase	Primary Endpoint	Follow-Up Period
AZALEA TIMI 71	-	The primary end point was major or clinically relevant nonmajor bleeding	22 months
OCEANIC AF	Phase 3	The primary efficacy objective was to determine whether asundexian is at least noninferior to apixaban for the prevention of stroke or systemic embolism. The primary safety objective was to determine whether asundexian is superior to apixaban with respect to major bleeding events.	12 months
PACIFIC AF	Phase 2	The primary endpoint was the composite of major or clinically relevant non-major bleeding according to International Society on Thrombosis and Haemostasis criteria, assessed in all patients who took at least one dose of study medication.	12 weeks

## Data Availability

All relevant data are within the manuscript and are fully available without restriction.

## Supplementary Materials

The following supporting information can be downloaded at: https://www.mdpi.com/article/10.3390/jcm14228234/s1, File S1: PRISMA CHECKLIST (2020) (Reference [18] has been cited in this file); Table S1: Risk of bias summary table.

## Abbreviations

The following abbreviations are used in this manuscript:

AF	Atrial fibrillation
OAC	Oral anticoagulation
DOAC	Direct oral anticoagulant
VKA	Vitamin K antagonist
FXI	Factor XI
FXIa	activated Factor XI (Factor XIa)
