# Supplementary material for: Balancing Safety and Efficacy: Factor XIa INHIBITORS vs. DOACs in Patients with Atrial Fibrillation: A Systematic Review and Meta-Analysis of Randomized Controlled Trials
Source: J Clin Med. 2025 Nov 20;14(22):8234. doi: 10.3390/jcm14228234 (PMC12653192; doi:10.3390/jcm14228234)
Supplement: Supplementary file 1 [file jcm-14-08234-s001.zip › jcm-3889807-Supplementary Table S1.pdf]

Supplementary Table S1: Risk of bias summary for randomized studies (RoB 2)

Study	Bias from the randomization process	Bias due to deviations from intended interventions	Bias due to missing outcome data	Bias in the measurement of the outcomes	Bias in the selection of the reported result	Overall risk of bias
AZALEA-TIMI 71	Low	Low	Low	Low	Low	Low
OCEANIC-AF	Low	Low	Some concerns	Low	Low	Some concerns
PACIFIC-AF	Low	Low	Low	Low	Low	Low
